# Evaluating a school-based intervention through routine local authority data and national school data: Challenges and opportunities.

**DOI:** 10.23889/ijpds.v7i3.1886

**Published:** 2022-08-25

**Authors:** David Westlake, Kim Munnery, Linda Adara, Lena Meister, Philip Pallmann, Stavros Petrou, James White, Fiona Lugg-Widger

**Affiliations:** 1 Cardiff University; 2 Oxford University

## Objectives

The Department for Education funded local authorities (LAs) in England to embed social workers into schools (SWIS) so that they can work more effectively with teachers, children and families. A two-arm cluster randomised-controlled trial is evaluating the effect of SWIS compared to schools without on referrals to Children’s Social Care.

## Approach

LAs collate information about all children who are referred to Children’s Social Care within their authority. A case report form has been developed by the trial team to collect school-level information on child protection enquiries, referrals, child in need assessments and numbers of days children spent in state care. Cost data are also included for an economic evaluation. LAs return a completed dataset each quarter, reporting aggregate numbers by school and year group. Data on attendance and attainment will be requested separately from the National Pupil Database (NPD) in 2023.

## Results

Quarterly (n=6) data was supplied for 291 schools across 21 LAs. We will focus on this method of data collection and its challenges and opportunities. Relying on 21 external parties to provide all trial data in a timely manner brought risks to the collation of trial outcome data but has reduced the overall costs of data collection. LAs work with a number of different (and changing) IT systems, many of which are not compatible with each other. This, along with personnel changes in data teams, has led to delays. Receiving aggregate data limits the amount of quality control possible, therefore detailed guidance mitigated potential data input errors. Working with LAs to develop and refine these guidance materials at the outset paid dividends.

## Conclusion and Relevance

Using routine LA data is a novel cost-efficient approach to collecting trial outcome data at scale. However, the disjointed and varying data architecture that exists in LAs presents practical challenges. This means we relied on good working relationships with the LAs, including regular communication and collaborative problem solving.

